# Supplementary material for: Complexome profiling of the Chlamydomonas psb28 mutant reveals TEF5 as an early PSII assembly factor
Source: Plant Cell. 2025 Mar 17;37(6):koaf055. doi: 10.1093/plcell/koaf055 (PMC12164590; doi:10.1093/plcell/koaf055)
Supplement: koaf055_Supplementary_Data [file koaf055_supplementary_data.zip › psb28_interactive.zip!/plots/intensity/qualityPlots/peptides/SSA10_Cre03.g201300.t1.1.html]

### Cre03.g201300.t1.1

Trivial name: SSA10  
SeqCoverage [median]: 3.30  
Mass: 67.67 kDa  
  
 

| Sequence | MissedCleavage | Mass [Da] | wtIntensity | muIntensity | Score |
| --- | --- | --- | --- | --- | --- |
| AGGSTQVALR | 0 | 958.52 | 310.74 | 1267.37 | 164.68 |
| GGQELSPLGTR | 0 | 1113.58 | 0.00 | 1301.02 | 93.11 |
|  |  |  | 310.7 | 2568.4 |  |
